# Supplementary figures and images for: InteracTor: Feature engineering and explainable AI for profiling protein structure-interaction-function relationships
Source: PLoS Comput Biol. 2025 Oct 13;21(10):e1013038. doi: 10.1371/journal.pcbi.1013038 (PMC12614802; doi:10.1371/journal.pcbi.1013038)

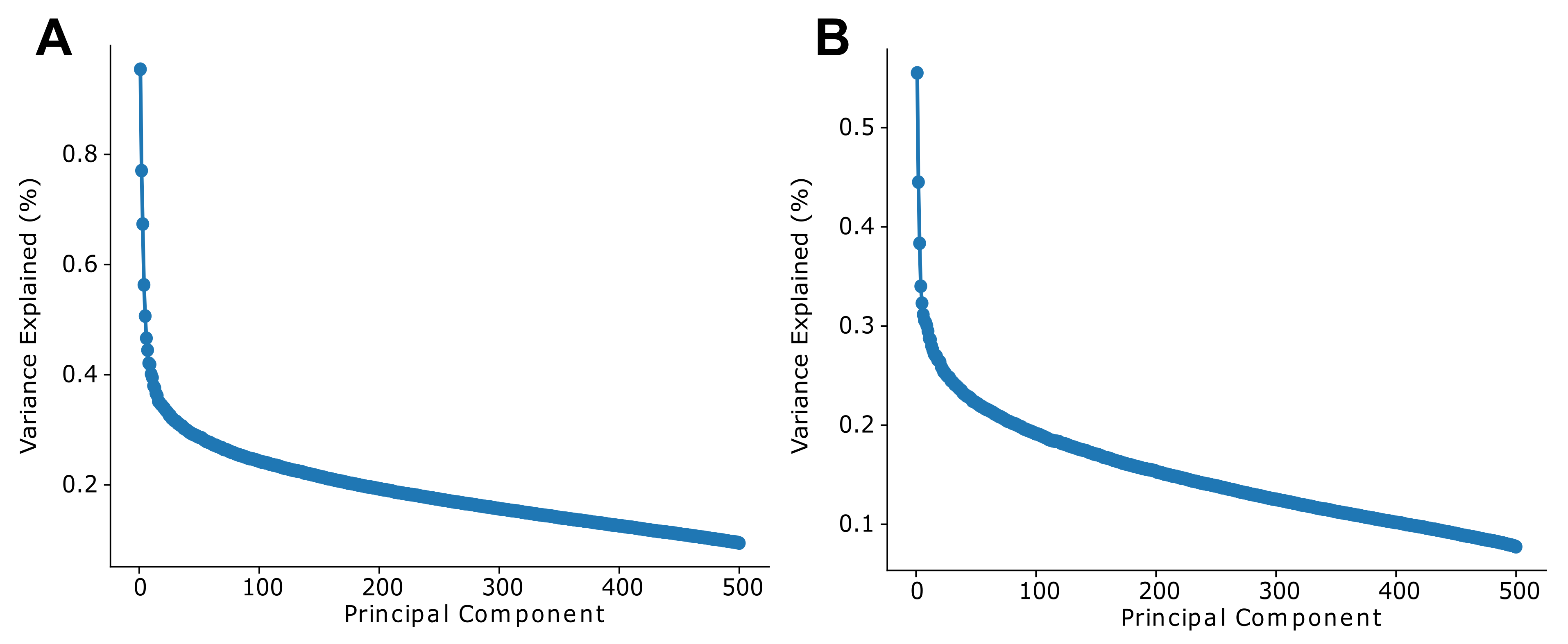

Supplement: S1 Fig — A) Scree plot illustrating the variance captured by each of the 500 principal components across different protein families. B) Scree plot depicting the variance explained by each of the 500 principal components across Gene Ontology (GO) terms. (TIFF) [file pcbi.1013038.s001.tiff]

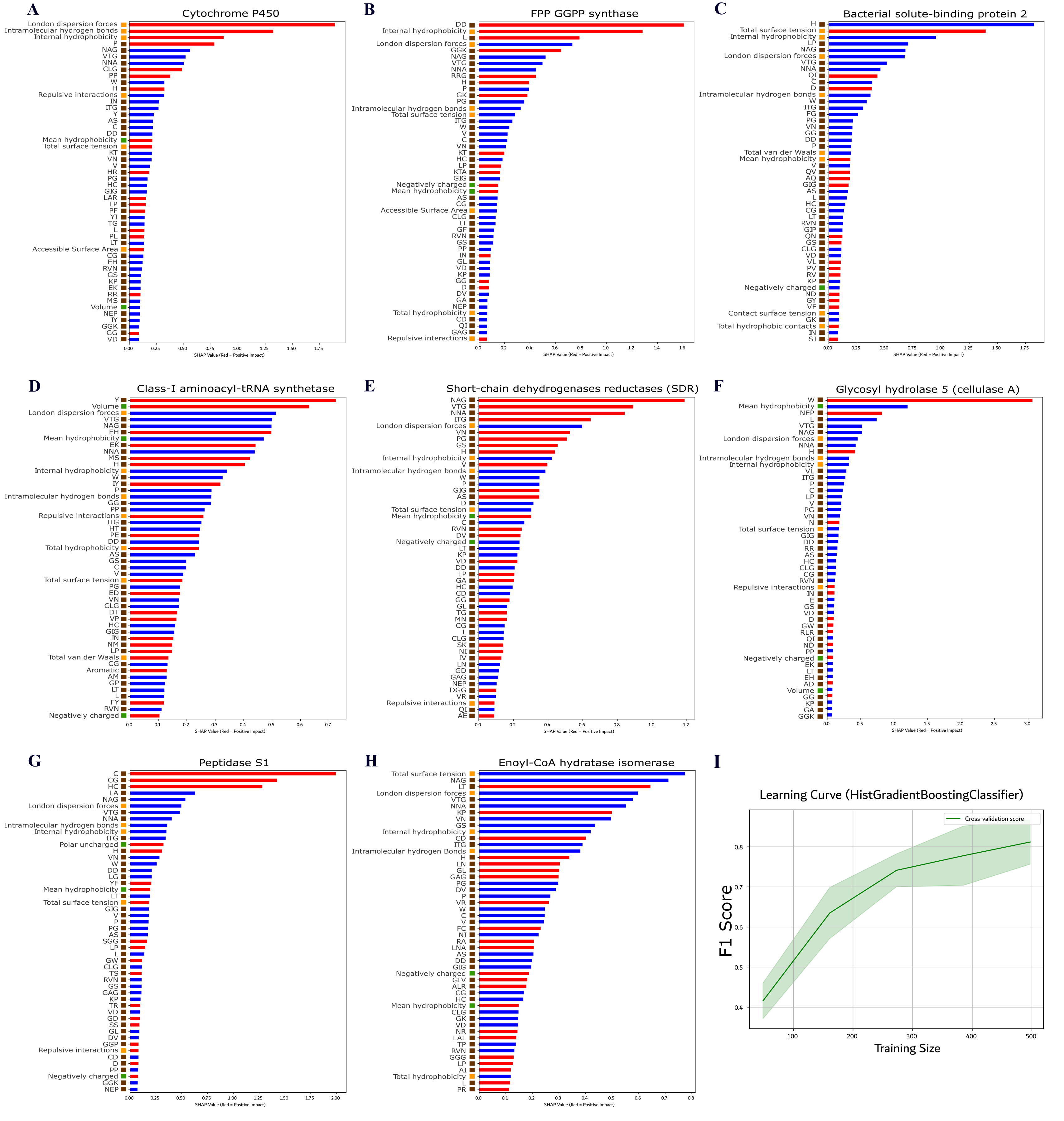

Supplement: S2 Fig — Mean absolute SHAP values were computed for each protein family, representing the overall impact of features on each protein family. The direction of the impact was computed based on the correlation between the SHAP values and the likelihood of the predicted class: red for positive correlation, blue for negative correlation. Subplots (A-H) correspond to distinct protein families. Colored tiles beside each feature indicate feature types: orange for 3D structural features (based on interatomic interactions and structural properties), green for CPAASC, and brown for sequence compositional (n-peptide) features. I) Learning curve for the best model (Histogram Gradient Boosting), showing validation F1 score as a function of training sample size. (TIFF) [file pcbi.1013038.s002.tiff]
