# Supplementary material for: InteracTor: Feature engineering and explainable AI for profiling protein structure-interaction-function relationships
Source: PLoS Comput Biol. 2025 Oct 13;21(10):e1013038. doi: 10.1371/journal.pcbi.1013038 (PMC12614802; doi:10.1371/journal.pcbi.1013038)
Supplement: S1 Table — (DOCX) [file pcbi.1013038.s003.docx]

**S1 Table**: Protein physicochemical features based on chemical properties of amino acid side chains (CPAASC).

| Feature Name | Feature Description |
| --- | --- |
| Non-polar aliphatic^80^ | Frequency of non-polar aliphatic residues in a protein, which is a key indicator of its structural stability and folding behavior, often signaling the presence of a hydrophobic core or membrane-spanning regions. |
| Aromatic^81^ | The frequency of aromatic residues (phenylalanine, tyrosine, and tryptophan) are keys in π-π stacking interactions. |
| Polar uncharged^82^ | The frequency of polar uncharged residues is important for hydrogen bonding with water and other polar molecules, contributing to protein solubility and interactions within proteins. |
| Positively charged^83^ | The frequency of positively charged residues incluences protein folding stability by forming salt bridges and other electrostatic interactions. |
| Negatively charged^84^ | The frequency of negatively charged residues contributes to protein folding stability and solubility through their ability to form ionic bonds and interact with positively charged molecules and ions in pH-dependent processes. |
| Mean molecular weight^85^ | The average molecular weight of amino acids is a parameter crucial for understanding and manipulating proteins and peptides for protein characterization, quantification, design, and metabolic studies [^86^]. Mean molecular weight of a protein, measured in Daltons (Da), which reflects the sum of the masses of all its constituent amino acids divided by the total residue count. |
| Mean volume^87^ | Refers to the three-dimensional space occupied by a protein molecule, measured in cubic angstroms (Å³), including both the atoms and any internal cavities or pockets, which are relevant for understanding protein packing, stability, and potential binding sites. |
| Mean hydrophobicity^88^ | Hydrophobicity plays is an essential element in protein folding, as hydrophobic amino acids tend to cluster together in the protein core, away from water. We calculate the mean hydrophobicity by assigning hydrophobicity values to each residue using Expasy’s ProtScale parameters, summing these values across the whole protein sequence and dividing it by the protein length^89^. |
