# Supplementary material for: InteracTor: Feature engineering and explainable AI for profiling protein structure-interaction-function relationships
Source: PLoS Comput Biol. 2025 Oct 13;21(10):e1013038. doi: 10.1371/journal.pcbi.1013038 (PMC12614802; doi:10.1371/journal.pcbi.1013038)
Supplement: S2 Table — (DOCX) [file pcbi.1013038.s004.docx]

**S2 Table:** Protein sequence composition features.

| Feature Name | Feature Description |
| --- | --- |
| Monopeptide composition:  A, R, N, D, B, C, E, Q, Z, G, H, I, L, K, M, F, P, S, T, W, Y, V, U (selenocysteine), O (Pyrrolysine) and Homocysteine | Frequency of individual residues in a protein. The monopeptide composition impacts protein folding, stability, and interactions (e.g., thermophilic proteins often have more charged residues for increased stability)^90^. |
| Dipeptide composition:  AA, AR, AN, AD, AC, AE, AQ, AG, AH, AI ... ZS, ZT, ZW, ZY, ZV, ZO, ZU | Frequencies of each possible pair of residues (k-mers where k=2) in a protein. The distribution of dipeptides varies among different protein structural classes (e.g., all-alpha, all-beta, alpha+beta)^91^, providing insights into common structural motifs across protein families. |
| Tripeptide composition:  AAA, AAR, AAN, AAD, AAC, AAE ... BBY, BBV, BBO, BBU, BBB | Tripeptide frequencies, extracted as k-mers with k=3, can highlight functionally important regions such as catalytic triads in enzymes (e.g., Ser-His-Asp in serine proteases)^91,92^. |
