# Supplementary material for: InteracTor: Feature engineering and explainable AI for profiling protein structure-interaction-function relationships
Source: PLoS Comput Biol. 2025 Oct 13;21(10):e1013038. doi: 10.1371/journal.pcbi.1013038 (PMC12614802; doi:10.1371/journal.pcbi.1013038)
Supplement: S3 Table — (DOCX) [file pcbi.1013038.s005.docx]

**S3 Table**: Distribution of selected protein families.

| Protein Family | Number of Proteins |
| --- | --- |
| Short-chain dehydrogenases/reductases (SDR) family | 196 |
| Cytochrome P450 family | 90 |
| Enoyl-CoA hydratase/isomerase family | 73 |
| Bacterial solute-binding protein 2 family | 41 |
| Class-I aminoacyl-tRNA synthetase family | 39 |
| Glycosyl hydrolase 5 (cellulase A) family | 37 |
| Peptidase S1 family | 37 |
| FPP/GGPP synthase family | 36 |
