# Supplementary material for: InteracTor: Feature engineering and explainable AI for profiling protein structure-interaction-function relationships
Source: PLoS Comput Biol. 2025 Oct 13;21(10):e1013038. doi: 10.1371/journal.pcbi.1013038 (PMC12614802; doi:10.1371/journal.pcbi.1013038)
Supplement: S4 Table — (DOCX) [file pcbi.1013038.s006.docx]

**S4 Table:** Distribution of selected gene ontology terms.

| Gene Ontology (GO) term by ligand type | Number of proteins |
| --- | --- |
| metal ion binding [GO:0046872, GO:0000166] | 473 |
| DNA or nucleotide binding [GO:0003677] | 278 |
| carbohydrate binding [GO:0030246] | 97 |
