# Supplementary material for: InteracTor: Feature engineering and explainable AI for profiling protein structure-interaction-function relationships
Source: PLoS Comput Biol. 2025 Oct 13;21(10):e1013038. doi: 10.1371/journal.pcbi.1013038 (PMC12614802; doi:10.1371/journal.pcbi.1013038)
Supplement: Attachment — Submitted filename: Response_to_reviwers_1.docx [file pcbi.1013038.s013.docx]

Dear Reviewers,

Thank you for all your insightful comments and suggestions. We have carefully addressed all comments and incorporated your suggestions into the manuscript. We believe that the manuscript has been improved and is now more robust. Below, we indicate all the modifications made to the manuscript. Thank you very much for your attention and for taking the time to review our manuscript.

Best regards,

Dr. Silva, et. al

Response to reviewers' comments:

**Reviewer #1**

The work presented here describes a predictive ML method for the structural classification of protein families and their function. Several aspects, namely the use of 3D structures and interpretable models, are two of the strengths of this work. Overall, the manuscript covers all the methodological details of the work with sufficient clarity, and approaches appear to be rigorous and robust.

I do have some minor reserves mostly related to the **results analysis** and the implications of the features deemed responsible of the model performance, and minor wording clarifications throughout the manuscript. In particular, the correlation between features role and protein classes feels rather anecdotal and week. This part could be strengthen very easily by finding more examples and consistent outcomes across protein families (i.e.: all/the majority of transporters show features X and Y).

We appreciate the feedback, we addressed this concern by further improving the section entitled "**Mechanistic insights on protein structure, activity, and function**" at pages 17 (lines 368-384).

Below a few more in-detail comments.

1) vdW vs London forces: interesting take, but how did they calculate them with sufficient accuracy to differentiate them and prevent double-counting?

Thanks for your question. We included further clarification in Table 1 (pages 6-7):

*“While the VDW feature considers interactions within a distance threshold of ≤ 0.7 plus the sum of the van der Waals radii, London dispersion focuses on longer-range interactions, thereby minimizing overlap between these features.”*

Further details and mathematical formulas are described in our previous article, so we included its citation in Table 1 as well (reference 5, page 25, lines 572-574):

*“Dias R, Kolazckowski B. Different combinations of atomic interactions predict protein-small molecule and protein-DNA/RNA affinities with similar accuracy. Proteins. 2015 Nov;83(11):2100-14. doi: 10.1002/prot.24928. Epub 2015 Sep 23. PMID: 26370248; “*

2) Out of the features extracted, how the Authors addressed the dominance of features describing the frequency of a tripeptide? While not being at risk of providing unwanted memorization of the dataset, it still represents a dominant set of features that capture the sequence more than the structural context of the amino acids, arguably one of the strengths of the work.

Yes, it is correct to note that, in this analysis, other features were more important than tripeptide descriptors according to Mutual Information. We addressed this question in Discussion section (page 14, lines 284-294).

*“Notably, several dipeptide-based descriptors, including TG, VN, and GG, were identified among the top-ranked features in our mutual information analysis. Given the much larger number of possible tripeptides compared to dipeptides, we would expect more tripeptides than dipeptides to appear among the top features, reflecting a feature representation bias. However, MI scoring inherently adjusts for feature frequency and only highlights those patterns that occur often enough to be reliably informative. This helps account for feature representation bias, so the overrepresentation of certain n-peptides among top predictors reflects their actual predictive value with the available sample size, rather than simply their greater number. Biologically, tripeptides capture more specific local sequence contexts and functional motifs that are crucial for molecular recognition, enzymatic activity, and binding interactions(36,37). Therefore, beyond statistical considerations, the prominence of some tripeptides among top features likely reflects their genuine molecular and functional importance.”*

In that regard, I assume that in the Mutual Information analysis, the descriptors of TG, VN and GG descriptors refer to the frequency of dipeptides of such amino acids. If that's correct, it's interesting that dipeptide descriptors were found more important than tri-peptides?

The point raised is valid: the sheer number of tripeptide frequency features, which surpasses other feature types by two orders of magnitude, can potentially skew a model’s focus toward sequence-derived information at the expense of structural or interatomic-interaction-based context. We address this issue (page 14, lines 284-294) and do not dismiss the value of sequence features. Instead, we emphasize that their main contribution lies in the integration of structural and interatomic interaction features along with sequence features.

3) Additionally, certain peptide composition patterns, such as those involving amino acids P, CLG, PP, H, and W, were also among the most important features. This is somehow a recurrent pattern in the manuscript, where amino acid one-letter references are mixed with other acronyms in a not so clear manner. Also, according to the nomenclature introduced earlier, those should be referred to as n-peptide descriptors, not "amino acids".
We standardized the nomenclature in the manuscript and across all figures and tables. The feature name is clearer now. Thank you for this suggestion.

4) For instance, the importance of London dispersion forces(45) and internal hydrophobicity(46) in Cytochrome P450 classification emphasizes the crucial role these interactions play in maintaining its tertiary structure. This statement and its inverse in the following paragraph are a bit controversial. Hydrophobicity is considered among the most important forces driving protein folding, which would be expected to hold true for virtually every protein in the dataset, and not in specific classes.

We further clarified our statement in the manuscript updating the “**Mechanistic insights on protein structure, activity, and function”** section: page 17, lines 369-384.

“*The features extracted by InteracTor provide critical insights into the structural and functional diversity of analyzed protein families. Hydrophobicity-related features consistently rank among the top predictors across most families; however, internal hydrophobicity is particularly important for classifying the Cytochrome P450 and FPP GGPP synthases, while mean hydrophobicity plays a more prominent role in other protein families (Fig. 6, Supplementary Fig. S2). The variation in feature importance can be attributed to the structural and functional diversity of the protein families. For example, the importance of London dispersion forces(49) and internal hydrophobicity(50) in Cytochrome P450 classification(51) emphasizes the crucial role these interactions play in maintaining its tertiary structure (Fig 6A). The tripeptides NAG and NNA showing as the top 1 and top 3 predictors in Short-chain dehydrogenases reductases (SDR) are consistent with the conserved NNAG motif, known for stabilizing the β-strands within the central β-sheet of the characteristic Rossmann fold that is critical for cofactor binding and enzymatic function in this family (Fig. 6E)(52,53). The tripeptides VTG and ITG showing as the top 2 and top 4 redictors are also consistent with the glycine-rich cofactor-binding motif (TGxGxxxG sequence) in the SDR family(52). These peptide features likely capture key conserved elements of the NAD(P)(H) binding site, reflecting their important structural and functional role in SDR enzymatic activity(54). These family-specific variations highlight how different feature types contribute distinctively and synergistically to protein classification.”*

5) Bacterial solute-binding protein 2 family displayed a balance of repulsive interactions and internal tension, likely linked to their dynamic conformational states required for substrate transport (54). This is an interesting finding, but unless confirmed across multiple dynamic proteins, it really falls a bit short of supporting the post-hoc analysis discussed.

We have improved the sentences following the suggestions around our affirmations (page 18, lines 394-398):

“…*The Bacterial solute-binding protein 2 family displayed a balance of total surface tension and internal hydrophobicity, showing equally intense but opposite effects (Fig. 6C), which could reflect in the dynamic conformational states involved in substrate transport (55)(56). While this finding is intriguing, further studies involving other dynamic protein families are necessary to confirm whether this behavior is a generalizable feature*.”

Also, as a suggestion for future work, it would be very interesting to see the performance of a model extended to include secondary structure descriptors.

We are grateful for the suggestion to incorporate the secondary structure descriptors in InteracTor in the future, which can be helpful to improve model performance.

**Reviewer #2**

This paper by Silva et al explores the classification of protein family and protein function using machine learning methods. The specific contribution of this work is the use of features based on 3D protein structures. These features are based on, for example, patterns of hydrogen bonding, hydrophobic contacts, and van der Waals interactions. Overall, this work is scientifically interesting and rigorously conducted. I have only three comments for the authors' and editors' consideration:

1) First, this work is based on the 20,877 protein structures in the PDB REDO data set. The manuscript provides good justification for using this subset of the entire PDB based on data quality and data leakage. But the thresholds used only leave 8 protein families and 3 GO terms, which limit the generalizability of the conclusions. Given that the GO analysis uses a higher threshold for the sample size (90 vs 30), the authors should consider relaxing that **threshold to increase the number of GO terms** in their analysis.

We thank the reviewer for their comment on the number of protein families and GO terms analyzed. We applied conservative filtering to prioritize data quality and interpretability over quantity. Because GO annotations are not mutually exclusive—a single structure protein can carry multiple terms across Molecular Function, Biological Process, and Cellular Component—indiscriminate inclusion would have inflated the dataset, introducing redundancy and noise. Due to the hierarchical and semantically overlapping nature of GO terms, expanding the set would have required extensive curation and additional validation beyond the study’s scope. In fact, one ongoing project in the lab specifically focuses on predicting GO terms for genes and proteins, which aligns well with this direction. We agree this is a valuable avenue for future work.

2) Second, Figure 5 shows the F1-score on subsets of features as they compare to the full set of features. It appears this evaluation was done based on a 80-20 split, where 80% of the samples are used to train the classifier and 20% are used to calculate the F1-score. I infer that the feature selection was also done on the 80% training set. I believe that the more rigorous approach would be a three-way split of samples, with one set used strictly for the feature selection. This design would better support the generalized assertion that the feature selection method produces comparable classification results.

Thank you for your insightful comment regarding the data splitting and feature selection procedure. We took additional care to avoid information leakage by ensuring that all feature selection and model optimization steps were conducted strictly within the training data (the initial 80%). The test data remained fully independent for evaluation purposes. We included this clarification in the Methods section (page 23, lines 525-526).

We agree that employing a three-way split can provide a more rigorous assessment of feature selection methods by preventing any potential information leakage from the feature selection step into the test evaluation. However, some protein family classes in our analysis are quite rare, with 3 families represented by fewer than 37 protein structures in the entire dataset. Implementing a three-way split would require us to further reduce the size of each subset. This would compromise the already limited sample size for these rare classes, undermining both statistical power and the reliability of model evaluation, especially for minority classes. Further reducing the available data for either training or testing would have required the exclusion of the most infrequent families in order to obtain sufficient power, restricting our ability to evaluate and compare model performance across a broad landscape of protein families. A reduced training set, or the removal of rare families, would also directly limit subsequent analyses, including the explainable AI analyses performed with SHAP values, since a minimum sample size per class is required to compute feature importances and generalization metrics. This would detract from the central goal of our work: a feature extraction tool to characterize protein structural and functional properties across diverse protein families.

Thus, while we recognize the theoretical advantages of the more conservative three-way split, in the context of our dataset's constraints and the objectives of high-coverage comparative modeling, we prioritized maximizing training data to preserve both statistical power and class diversity. This compromise follows established practice in similar high-dimensional, multi-class machine learning studies with rare classes.

3) Third, in Figure 6, consider highlighting the 3D interaction features (by bolding or coloring) that are the focus of this study. Especially since the feature lists are heavily dominated by the compositional features, visually highlighting those interaction features will reinforce their importance.

We color coded the feature types in Figure 6 and Supplementary Figure S2. To assign a statistical significance, consider also using a Kolmogorov-Smirnoff statistic to show that these interaction features have larger SHAP values (similar to how the GSEA tool performs gene set enrichment).

The Kolmogorov-Smirnov tests were very helpful to identify interatomic interaction features that exhibited significant enrichment. We are glad to include your suggestions in the manuscript. Please see the Results (pages 12-13 lines 249-259), Discussion (page 17, lines 358-366) and Methods section (pages 23-24 lines 541-556).
